# Quadriostial Origin of the Coronary Arteries: A Case Report

**DOI:** 10.7759/cureus.80653

**Published:** 2025-03-16

**Authors:** Hunter Simonsen, Kaden Taylor, Luke Brogan, Thaddeus Reed, Taylor Geddes, William Craig

**Affiliations:** 1 Medicine, Kansas City University of Medicine and Biosciences, Joplin, USA; 2 Cardiology, Mercy Hospital, Joplin, USA

**Keywords:** anomalous left circumflex artery, cath lab, coronary artery anomaly (caa), first obtuse marginal branch of the left circumflex artery, four coronary ostia, left anterior descending artery (lad), left heart catheterization, right coronary artery (rca), sudden cardiac death (scd)

## Abstract

A 75-year-old male, with a history of hypertension and peripheral vascular disease, presented to the cardiac catheterization lab for an elective coronary angiogram after an abnormal cardiac stress test. The coronary angiogram results showed a total of four separate coronary artery ostia. Within the right coronary cusp, there were two separate ostia as opposed to a single ostia that supplies the right coronary artery (RCA). The additional ostia gave rise to the left anterior descending artery (LAD). Further, as opposed to the single ostia in the left coronary cusp, there were two separate ostia creating a left circumflex (LCX) and marginal artery. Despite this anomalous anatomy, the patient was asymptomatic and instructed to continue to follow up with outpatient cardiology. Our case report discusses this unique quadriostial coronary anatomy, the complications that can arise from anomalous coronary arteries, diagnoses, and management of such cases.

## Introduction

Anomalies of the coronary arteries are relatively rare and have been discovered in various ways. As in our case, many are found incidentally, either during autopsy or through routine coronary angiograms [1]. While many anomalies are clinically silent and determined to be benign, others can contribute to an increased risk of acute coronary syndrome (ACS), sudden cardiac death (SCD), and life-threatening arrhythmias [2].

Below is a unique finding of a quadriostial anomaly of the coronary arteries found in an elective coronary angiogram. The right coronary artery (RCA) and left anterior descending artery (LAD) originate from their own individual ostia in the right coronary cusp, a rare finding reported at around 0.15% of coronary artery anomalies (CAA) [3]. The left circumflex (LCX) and marginal branch also had their own separate ostia originating from the left coronary cusp. This is compared to the typical CAA, comprised of two coronary artery ostia: The left coronary ostia, located in the left anterior aortic sinus, gives rise to the LCA. The right coronary ostia, located in the anterior aortic sinus, gives rise to the RCA. Post-angiogram workup consisted of further imaging with CTA to effectively rule out interarterial or retroaortic courses of the LAD, which are associated with poor outcomes [2]. Following confirmation, a regular follow-up was scheduled to monitor for any worsening symptoms.

## Case presentation

**Figure 1:**
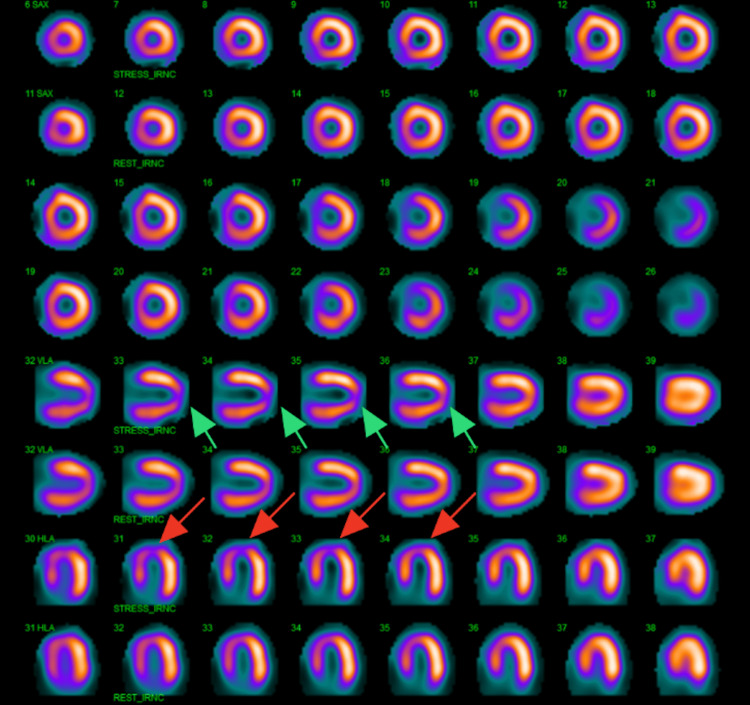
Nuclear stress test showing signs of medium-sized, moderate-intensity, reversible perfusion defect involving the distal anteroseptal wall and apex. Arrows indicate the areas of ischemia in the vertical long (green) and horizontal long axis (red).

**Figure 2:**
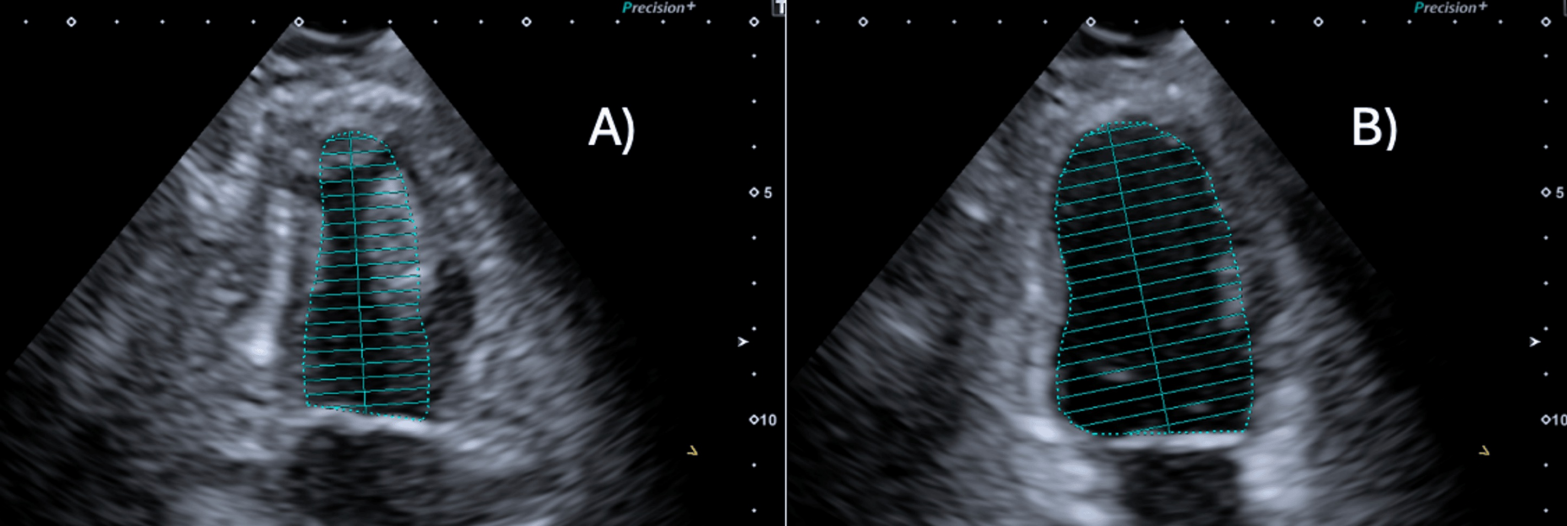
Images of the echocardiography done prior to the procedure showing an overall computed ejection fraction of 65%. A) Image of the left ventricle in full systole. B) Image of the left ventricle in full diastole.

A 75-year-old male, with a past medical history of penile squamous cell carcinoma in remission, chronic hypertension, and peripheral vascular disease (PVD), presented to the hospital for an elective left heart catheterization after two months of worsening shortness of breath with exertion. He was referred to cardiology by his oncologist to rule out cardiogenic causes of his shortness of breath. Due to the patient's history of radiation-induced PVD and subsequent placement of femoral stents, a nuclear stress test was ordered to rule out any cardiac ischemia. The nuclear stress test revealed a medium-sized, moderate-intensity, reversible perfusion defect involving the distal anteroseptal wall and apex (Figure 1). An echocardiogram (ECHO) was done three days prior to the procedure. It showed an ejection fraction (EF) of 65%, moderate left ventricular hypertrophy (LVH), and ascending aorta dilation of 39.0 mm compared to the normal 23.0 mm (Figure 2). Further, it is concluded that this normal value is subject to change as age, gender, and body size can lead to increased values [4]. His medications before the procedure included losartan 100 mg daily and ezetimibe 10 mg daily.

**Figure 3:**
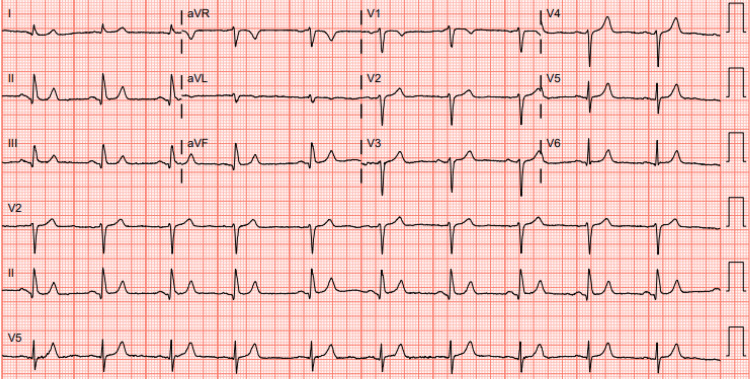
Patient ECG shows normal sinus rhythm with a heart rate of 62 beats per minute done on the day of left heart catheterization. ECG: electrocardiogram

On the day of his left heart catheterization, he was hypertensive with a blood pressure reading of 177/92. His pulse rate was 79 bpm, respiratory rate was 20 per minute, and oxygen saturation was 100% on room air. An electrocardiogram (ECG) on the day of the procedure read normal sinus rhythm and was otherwise unremarkable (Figure 3). Routine complete blood count (CBC), basic metabolic panel (BMP), and lipid panel were also drawn. The results of these lab draws are portrayed in Table 1. The patient was hemodynamically stable and agreed to proceed with the procedure with possible intervention.

**Table 1:** Results from the CBC, BMP, and lipid panel on the day of left heart catheterization. BMP: basic metabolic panel; BUN: blood urea nitrogen; CBC: complete blood count; GFR: glomerular filtration rate; HDL: high-density lipoprotein; LDL: low-density lipoprotein; RBC: red blood cells; WBC: white blood cells

Items	Reference Ranges & Units	Patient Values
CBC		
WBC	4.0-11.0 K/uL	7.1
RBC	4.70-6.00 M/uL	4.76
Hemoglobin	13.5-18.0 g/dL	14.9
Hematocrit	42.0-52.0%	43.9
BMP		
Sodium	136-145 mmol/L	136
Potassium	3.5-5.1 mmol/L	4.0
Chloride	98-107 mmol/L	98
CO_2_	22-29 mmol/L	23
Calcium	8.8-10.2 mg/dL	9.6
BUN	8-23 mg/dL	13
Creatinine	0.67-1.17 mg/dL	0.93
GFR	>60 mL/min/1.73 m^2^	>60
Glucose	74-99 mg/dL	87
Lipid panel		
Cholesterol	<200 mg/dL	243 (H)
HDL	40-59 mg/dL	43
LDL Calculated	<100 mg/dL	173 (H)
Non-HDL Cholesterol	<130 mg/dL	200 (H)
Triglyceride	<150 mg/dL	137

**Figure 4:**
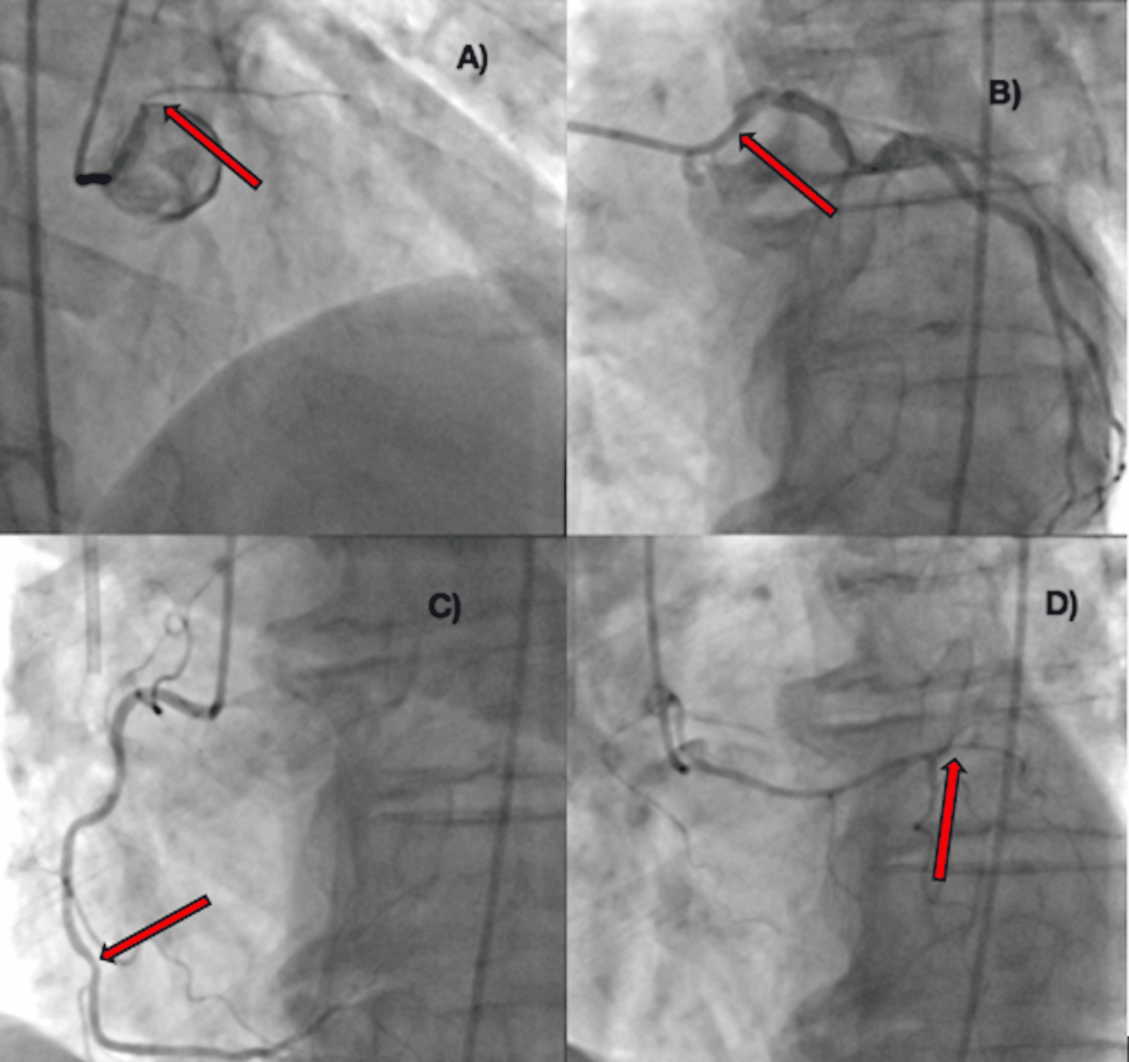
Coronary angiograms from the procedure showing four distinct coronary ostia. Red arrows indicate areas of stenotic lesions. A) The marginal branch is located on the left coronary cusp and is slightly superior to the left circumflex (LCX) - mild stenosis at the proximal portion of the branch. B) The left circumflex (LCX) is also located on the left coronary cusp with a 50% stenotic lesion in the proximal portion. Located inferior to the marginal branch. C) The right coronary artery (RCA) originates from its anatomically correct location and has a mild 30% stenotic lesion in the middle portion of the artery. D) The left anterior descending artery (LAD) with its ostium located in the right sinus of Valsalva on the right coronary cusp with its septal branches running inferiorly. A mild 40% stenotic lesion is observed in the middle portion of this artery.

After the left heart catheterization, it was determined that there were four distinct coronary ostia - all with mild atherosclerotic lesions. The right coronary cusp was found to have the RCA originating from its anatomically typical location and the LAD from its own ostia, in an anomalous location just inferior to the RCA. The left coronary cusp had an anomalous two ostia giving rise to a marginal branch and LCX artery. The ostium of the marginal branch originated from the superior portion of the left coronary cusp with the ostia of the LCX located slightly inferior. The marginal branch had mild stenosis at the proximal portion. The LCX had a 50% stenotic lesion in the proximal portion. The RCA had a mild 30% stenotic lesion in the middle portion of the artery. Finally, a mild 40% stenotic lesion was observed in the middle portion of this artery (Figure 4). These mild stenotic lesions were determined to be caused by lifestyle choices, including, but not limited to, exercise, diet, and tobacco use. They also correlated with what was shown on the nuclear stress test before the procedure. At this time, these lesions were under the 70% threshold indicated by the American Heart Association (AHA) guidelines for stent placement - although this does not negate the possible future need for intervention. We also concluded that further clarification of the course of the LAD would be safer for the patient before any intervention was performed. Therefore, percutaneous intervention (PCI) was deferred.

**Figure 5:**
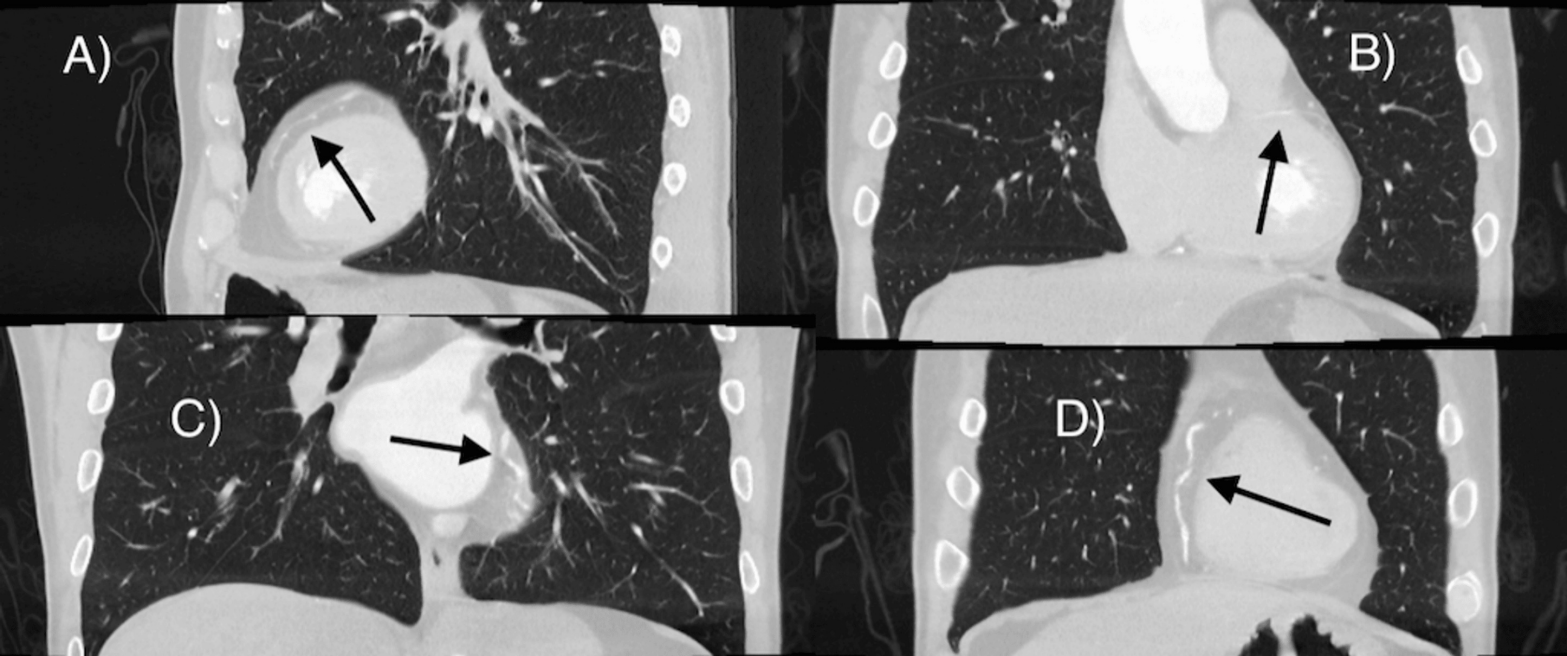
Computed tomography angiography of the heart and arteries. A) Anterior pathway of the left anterior descending artery (LAD) with septal branches running inferiorly. B) Marginal branch with mild proximal stenosis running laterally and then inferiorly. C) Left circumfle (LCX) is running inferolateral and originates from the left coronary cusp. D) Right coronary artery (RCA) running separately along the medial aspect of the heart with minimal stenosis.

Although PCI was not indicated, further follow-up was necessary to negate any increased risk of SCD. It was indeterminate at this time whether the LAD had a retroaortic course which could increase the likelihood of further complications and ischemia. Post-procedure, a CTA of the heart and arteries was performed to verify the path of the LAD. The CTA confirmed the presence of four coronary ostia and confirmed that the LAD was not compressed at the origin and coursed anteriorly to the aorta before traveling in the interventricular groove (Figure 5). The treatment plan due to the mild-to-moderate stenotic lesions was to add daily aspirin 81 mg and metoprolol tartrate 25 mg twice daily to his daily medications to help prevent further cardiac stress by reducing platelet aggregation and as an empiric treatment for lowering blood pressure. Although elevated cholesterol was evident in labs, any additional medication (i.e., statins, bile acid resins, fibrates) was delayed at this time. Further, the patient was scheduled for regular follow-up every six months to monitor his anomaly and cardiac symptoms with cardiac stress tests and echocardiography. Optional heart catheterizations were also recommended if any worsening chest pain or shortness of breath were to occur. If necessary, PCI would be performed during this time.

## Discussion

The prevalence of CAA, as well as the severity of the anomaly, varies widely between sources. Estimates utilizing invasive coronary angiography have reported ranges around 1%-6% [5] and 0.78%-1.3% [6]. However, studies using coronary computed CTA (CCTA) reported ranges between 0.99%-5.8% [7] and 0.2-1.3% [1]. The LAD and RCA originating from the right coronary sinus found is one of the rarest anomalies with an incidence rate of 0.15% [2]. As in our case, most cases of CAA are clinically silent and incidentally found on imaging or even at autopsy. More extreme anomalies such as tetralogy of Fallot and transposition of the great arteries infamously present acutely in infancy, while other various anomalies have been found in cases of angina, myocardial infarction, congestive heart failure (CHF), and cardiomyopathy. In cases of SCD, the second highest cause was noted to be CAA, with only hypertrophic cardiomyopathy accounting for more cases [8].

The AHA guidelines advise that the diagnoses of CAA be made with coronary catheterization with angiography, CCTA, or cardiac magnetic resonance angiography regardless of severity. These diagnostic imaging methods may be utilized following an abnormal ECG or cardiac stress test. However, in cases of incidental CAA findings, subsequent workup should be done to exclude tissue ischemia and arrhythmias [6]. Workup for ischemia may include the aforementioned ECG and cardiac stress testing, though nuclear perfusion imaging has proven to show the highest sensitivity concerning ischemia detection [9,10]. Ruling out specific high-risk anomalies such as an interarterial course of the LAD, high take-off of the coronary arteries, and slit-like ostia formation is also recommended [2].

Treatment of CAAs depends on the severity of the anomaly and whether or not tissue ischemia is detected. For individuals with high-risk anomalies, corrective surgery is recommended [10,11]. In older individuals with concurrent atherosclerosis of coronary arteries, PCI or coronary artery bypass grafting (CABG) may be recommended to ensure proper coronary perfusion. There are several surgical procedures available if corrective treatment is indicated, each with associated risks and benefits. These procedures include but are not limited to, unroofing, pulmonary artery translocation, reimplantation (ostial translocation), ostioplasty, and bypass grafting [11]. Unroofing can be considered when a patient with a CAA has an interarterial and intramural course [12]. When an anomalous vessel is being compressed between the pulmonary artery and the aorta, the pulmonary artery translocation approach can be considered. Reimplantation or ostial translocation can be chosen when the anomalous vessel does not course intramurally, or has a minor intramural course, with two separate ostia [13]. The ostioplasty approach is where the anomalous vessel is placed in the sinus that it would originally have exited by creating a new ostium [11,14]. A bypass grafting has a minor number of settings in which it is considered; specifically in patients with prior atherosclerotic narrowing, a bypass graft may be the surgical approach. Those undergoing a surgical procedure to address a CAA should understand the short-term and long-term risks and benefits and have a thorough conversation with their medical team. However, unroofing is the most common procedure used there is currently no recognized guideline by the AHA for a preferred surgical technique [15,16].

Non-surgical treatment may also be considered in patients with less severe CAA. A 5.6-year longitudinal study conducted in Japan reported efficacy in treating patients with CAA with medicine and restricting physical activity. Medication classes, such as nitrates, calcium channel blockers, beta-adrenergic blockers, and antiarrhythmic, were effective in preventing fatal cardiac complications, specifically in patients over the age of 50 without significant atherosclerosis [17]. In asymptomatic patients, poor surgical candidates, or with a negative stress test pharmacological treatment with or without activity restriction can be an effective option [18,19]. The decision between surgical and non-surgical intervention should be made with careful consideration of the patient's age, symptoms, specific anomalies, cardiac risk factors, and stress testing results.

Regardless of surgical or non-surgical intervention, management of patients with known CAA typically requires long-term follow-up. Depending on the severity of the CAA and the determined risk to the patient, routine ECGs, echocardiograms, and stress tests can be utilized by providers to monitor patients. The timeline for regular testing is case-specific and ensures that any changes in heart function or symptoms are detected early to minimize risks [11].

## Conclusions

This case demonstrates an example of a quadriostial origin of the coronary arteries found incidentally in a 75-year-old patient. Diagnosis of the condition was made through a coronary angiogram and confirmed with CTA. Both of these imaging modalities confirmed a less severe course of the LAD with low-risk potential for life-threatening complications. The decision on how to manage this patient was then based on his age, lack of severe atherosclerosis, symptoms, course of the LAD, and results of stress testing. Ultimately, a pharmacologic approach to treatment was chosen after extensive discussion with the patient. Regular biannual follow-ups with longitudinal stress testing and echocardiography were scheduled. Heart catheterization was also recommended for any worsening chest pain or shortness of breath.
